# The Paradox of Intervening in Complex Adaptive Systems: Comment on "Using Complexity and Network Concepts to Inform Healthcare Knowledge Translation"

**DOI:** 10.15171/ijhpm.2018.05

**Published:** 2018-01-24

**Authors:** Jacqueline Chandler

**Affiliations:** Cochrane, London, UK.

**Keywords:** Complex Adaptive Systems, Complexity Theory, Knowledge Translation

## Abstract

This commentary addresses two points raised by Kitson and colleagues’ article. First, increasing interest in applying the Complexity Theory lens in healthcare needs further systematic work to create some commonality between concepts used. Second, our need to adopt a better understanding of how these systems organise so we can change the systems overall behaviour, creates a paradox. We seek to manipulate systems that self-organise and follow their own internal rules. Although, our actions may impact and indeed meet some of our objectives, system behaviour will always emerge with unpredictable consequences. Likewise, outcomes at the aggregated level of the system never reaches an optimal point as defined by the ‘external controller.’ Kitson and colleagues’ theoretical model may struggle to resolve the paradox of gaining control over the multiple knowledge translation (KT) systems covered by the model, because theoretically these systems retain control under the principle of self-organisation. That is not to suggest that individual agents cannot influence system dynamics just that the desired outcome cannot be guaranteed. Indeed, for systems to change they will need strong incentives.

## Introduction

Applying Complexity Theory concepts is largely selective and individually interpreted. Many examples within healthcare^1-4^ are emerging as authors seek to make sense of how we manipulate Complex Adaptive System behaviour. Typically, core components^5^ that characterise the use of Complexity Theory are: Interaction, emergence, self-organisation, dynamic and nonlinear, feedback loops, sensitivity to initial conditions, etc. Kitson and colleagues have utilised some of these concepts in their model which prospectively designs knowledge translation (KT) initiatives and their subsequent evaluation. They shift the typical KT metaphors from pipeline and cycles to one of interactive and dynamic systems. KT is conceptualised as a “multidimensional, dynamic, complex integrated process.”^6^ Complexity thinking continues to strive to gain a foothold in healthcare.^7^ So, I welcome this theoretical development that continues the shift in thinking because much energy is often exerted into ineffective KT.^8^ In this commentary I make two key points, first, we need to be more systematic when applying this theoretical lens, and second, we need to consider the paradox created between the theory and our intentions.

## Defining Complex Adaptive Systems

Kitson and colleagues undertook an inductive approach to identify Complexity Theory concepts using selected key documents. Applying this conceptual shift in thinking they re-interpreted mechanistic metaphors, ‘bridging gaps’ and ‘pull- push’ to “synapses of interaction and connectivity.” Kitson and colleagues identify the core key KT Process steps and apply the “identified literature” of Complexity Theory to knit together their model. Following consultation workshops, they affirm and iterate their model. They tested the model retrospectively on two case study examples. This led to “further interrogation of the complexity and networking literature.” Although references are available this interrogation is not reported. As the Complex Adaptive Systems and Complexity Theory literature increases more systematic approaches are needed to justify theory building,^9^ the use and common understanding of key terms^10^ and the intersection with philosophy and other theories to understand system behaviour.^11^

The literature on Complexity Theory is extensive^5^ and increasingly applied to health and social systems. Different philosophical standpoints are taken such as complex realism^12^ and logical positivism,^13^ and connectionism.^14^ Careful application of self-organisation, emergence and complex adaptive systems notions need to take account of whether they address complex physical processes (weather or climate), biological systems (evolution) or social systems (human created structures). The theory is itself multi layered based on the type of, or system level observed with blurring between concepts used.^4^ Kitson and colleagues briefly define their terms, however, their application in the paper is at times inconsistent. We need to take care with the terminology and how we apply it to our setting or context of interest. The following definition, for example, blends physical and social systems.

“A Complex Adaptive System is a collection of diverse parts interconnected such that the organisation (or organism) grows over time without centralised control….CAS is generated by the adaptive interactions of its components (nodes, hubs, clusters).”^6^

## The Paradox of Controlling Self-organising Systems

Our models are abstractions of the real world and will only ever provide a partial representation. The purpose of the KT model is to distribute knowledge advocating a connectionist approach, however, Complex Adaptive System’s develop in such a way that system information is distributed throughout the system.^14^ That is each agent will only ever have a partial view of the whole system functioning. This creates a paradox between agents distributing knowledge between the connections of hubs and nodes by not having an overall view of that knowledge system and its multiple evolutions. Mechanisms such as audit can provide feedback on system behaviour at specific times. Likewise, greater connection between agents and their involvement potentially promotes distribution and take up of knowledge, however, in Complex Adaptive Systems, feedback between agents can either amplify or dampen the flow or connection of knowledge throughout the system. For human agents, this is typically their attitudes and beliefs. Increasing connections, as suggested, may be limited by the systems imperatives. For health systems, this is usually the busyness and pressure to meet priorities that will restrain adoption of new activity. System imperatives did not particularly feature in the paper. I would suggest this is an important driver in healthcare.^5^ The success of the London Atlas of Dental Development and Eruption could also be due to the strong imperative to resolve, in this instance, an urgent and tragic problem involving a major incident. Thus, this was possibly a strong imperative to gain involvement. Much knowledge dissemination does not necessarily have such a strong imperative, and other system imperatives may override.^5^

## Simple Rules and Incentives

Kitson and colleagues propose the KT Complexity Network will “generate the guiding principles or ‘simple rules’ required for the CAS to operate.”^6^

They suggest we can change the system rules once we have the model in place. First, the notion of ‘simple rules’ evolving into Complex Adaptive Systems needs greater explanation. John Holland captures the nature of Complex Adaptive Systems and the ‘simple rules’ that evolve into complex higher level aggregated system behaviour using examples as diverse as the immune system and the economy.^15^ He defines the three key characteristics of such systems in an early paper as evolution, aggregate behaviour and anticipation. To adapt and learn you need to anticipate, thus creating the rules to maintain the system. He suggests models of Complex Adaptive Systems are hard to create. However, he suggests we need to look at the distributed, rule based structure of these systems, as Kitson and colleagues seek to do with their model.

Holland describes these systems as undergoing continual change revising their rules and the system parts are each needing to adapt as they feed up to the aggregated structure that in turn feeds down. “As a result, the aggregate behavior of the system is usually far from optimal, if indeed optimality can even be defined for the system as a whole. For this reason, standard theories in physics, economics, and elsewhere, are of little help because they concentrate on optimal end-points, whereas complex adaptive systems ‘never get there.’ They continue to evolve, and they steadily exhibit new forms of emergent behavior”^15^ (p 20).

The perpetual motion of these systems overtime presents challenges to identification of the rules that create aggregated structures, which overlays the nodes and hubs in the network. Similarly, to create change or influence the aggregated structure requires finding ways to incentivize components (human agents) within the system allowing for the new emergent behaviour.^5^ Strategies are suggested to incentivize KT Networks with academic rewards. Complexity of human behaviour is an added dimension when applying the Complexity Theory lens. Others have sought to explain the emergence of social behaviour and structures.^12-14,16^ Conversation as an organising system with in human structures is one model advocated.^16^

Kitson and colleagues suggest that application of the KT Complexity Network requires ‘painstaking work,’ which will compete with other system incentives such as the need to maintain priorities within healthcare. Strumberg proposes whole health system transformation shifting the focus from a disease based system to health based, patient centred system that would operate using different rules (see Table, 21.2, p 252-54).^17^ However, although not yet accomplished, there is consistency of understanding across interpretations. I would suggest that social Complex Adaptive Systems are dynamic in their mobility, connectivity and evolution and are perpetually moving and developing. They retain parts of the original system structure overtime. However, through responsive adaption, gradual shifts and modifications they change. So, I suggest we ascertain the systems underlying organising rules as it moves from one state to another to tackle challenges it encounters, as well as meet the imperatives for its survival.

## Conclusion

Kitson and colleagues provide an account of how the KT Complexity Network model could be operationalised. I suggest their approach gets caught in the paradox of trying to control and change the behaviour of self-organising Complex Adaptive Systems. We need to understand the ‘rules’ that lead to the aggregated behaviour of the system rather than develop new rules for the system. Therefore, the focus should be on identifying the systems incentive or imperative to encourage system adaption.

## Ethical issues

Not applicable.

## Competing interests

Author declares that she has no competing interests.

## Author’s contribution

JC is the single author of the paper.
